# The Medico-Chirurgical Society of Edinburgh

**Published:** 1888-07

**Authors:** 


### FOREIGN MEDICAL SOCIETIES.

### THE MEDICO-CHIRURGICAL SOCIETY OF EDINBURGH.

ANIMAL TUBERCULOSIS IN RELATION TO CONSUMPTION IN MAN.

Thomas Walley, M. R. C. V. S., of the Edinburgh Veterinary College, said: In spite of all the attempts which have been made in the past to minimize the importance of animal consumption in its relation to this fell destroyer of the human race, the fact still remains that in every essential particular the tuberculosis of man and the tuberculosis of animals are identical. The scourge of the bovine species is the curse of the human, as it is also the pest of the feathered tribes.

A few years ago, comparatively, animal tuberculosis received but a scanty share of attention from the members of the veterinary profession in this country, and few in its ranks associated the malady with that most fatal and most intractable of all the diseases to which human flesh is heir; and, whether from the supineness exhibited by the members of the sister profession on this subject, or from the growing enlightenment of the members of the veterinary profession, I cannot say, but assuredly the latter have within the last twenty years taken the most prominent part in bringing the subject of tuberculosis under review.

In glancing for a brief space at the history of the disease, I need scarcely remind you, I think, of the fact that it is almost co-existent with the science of medicine; and probably, if we could look into the ages that were, we should find that its genesis was coeval with that of man.

If it is a fact that consumption in man has always held a prominent place in the literature of the medical profession since medicine became an art, it is equally a fact that the disease has been recognized by various cognomens among veterinarians for generations and in different countries as being common to animals.

But while English veterinary practitioners and others spoke glibly of "grapes" in cows, or, referring to a well-known symptom (diarrhœa) as "shooters," and to another symptom (sub-maxillary œdema) as "wattles," and while the characteristic growths of scrofula and tubercle were in many of the northern counties of England and in some of the Scottish counties designated and known as "clyers" and "wens"; and further, while the disease was known as "angleberries," and the affected animals themselves spoken of as "piners" or "wasters," still no systematic inquiry into its nature was made by those responsible for veterinary teaching until within the last quarter of a century. In proof of this I may quote the fact that during his admirable course of lectures—the most complete of any that were delivered at that time—Professor Simonds, of the London Veterinary College, did not once refer to the disease during my pupilage in the years 1861–3; and in 1847 there occurs in the professional organ of that day, the *Veterinary Record*, the following remarkable statements, in connection with some comments on what was evidently a case of nodular tuberculosis, which had been brought to the notice of the editor by a practitioner, Mr. W. Cox, of Ashbourne. The statements are: "The tumors attached to the viscera of the thorax of a cow are similar to some we have occasionally met with"; and, "On this subject our English veterinary authors are silent, and we believe the above to be the only case of the kind recorded."

In 1872 I had the privilege of reading a paper on this subject at a meeting of the West of Scotland Veterinary Medical Association, which was held in Glasgow; and again, in 1879, I included the disease in the book entitled *The Four Bovine Scourges*. On various occasions since the issue of that book I have brought the matter before the profession. During the period mentioned, other members of my profession have written upon and publicly noticed this subject, and for some years there has been, practically, unanimity among us as to the danger of transmission of the affection from animals to man. On the Continent the dangerous nature of tuberculosis was recognized at a very much earlier period than here, and we are told (*vide* Lydtin, Fleming, and Van Hertsen, on "The Influence of Heredity and Contagion in the Propagation of Tuberculosis") that in the Mosaic laws special provision was made for the condemnation of the flesh of animals suffering in the advanced stages of phthisis.

The promulgation of the opinion by Laennec that the existence of tubercles was always associated with a caseous centre in some organ or other of the body, was a vast stride towards the realization of its exact causal relationship. Then came the declarations of Villemin, founded on extended experimental inquiry as to its infectious character; and following on this, I think I am correct in saying, that the doubt cast upon the nature of the disease, in animals at least, by the use of the term lympho-sarcoma by Virchow, was the means of temporarily arresting the progress of the realization of an exact knowledge of its pathology, and, I may say also, of checking the realization of the intimate relationship existing between the tuberculosis of man and animals.

In Cohnheim's celebrated dictum, "That that which is of tubercular origin can alone originate tubercle," we had sounded the key-note of a great discovery; but it again was obscured to some extent by the declaration of Sanderson and others, that tuberculosis could be produced by inoculation with many and diversified pathological products. It remained for Koch to place the crown upon the edifice of our knowledge as to the specific nature of tuberculosis, though even his grand work had been foreshadowed to some extent by other inquirers. So far as I am personally concerned, I arrived at the conclusion at an early period of my professional career, *that tuberculosis was a specific disease;* and in the essay prepared in 1872 occurs the following remark: "Tubercle is as much a specific disease as is syphilis, or any other affection depending for its continuance (existence) in the system upon a morbid poison or principle." Again, in *The Four Bovine Scourges*, p. 150, "In whatever way I consider the character of tubercle as tubercle my mind always reverts to the same conclusion, viz., that tubercle is as much a specific disease, whether inherited or acquired, as glanders, syphilis, or any other affection of the kind with which we are acquainted."

Long before Koch's discovery, or rather his recognition of the tubercle bacillus, veterinary surgeons were acquainted with the fact that the tubercular nodules, so universally met with in the lungs of sheep, were simply a pathological expression of the effects of a parasitical irritant in the shape of the embryo of the Strongylus filaria; and it would have only been a logical sequence had some one who had carefully considered the matter arrived at the conclusion that the cause of tubercle as tubercle was an irritant; that that irritant was an organized entity in the shape of a bacillus, or that the irritant had some connection with such an organism—a secretion or product thereof, perhaps; that the irritation so produced established a localized exudative inflammation followed by a formative process, which, however, only attained to a certain degree of perfection; and, in consequence of the continued irritation and of the imperfect nature of the cell structures produced, that it was followed by a retrograde change which culminated in the recognizable caseous and calcareous products with which we are all so familiar.

Not only would such a line of reasoning have led us to the discovery of the true nature of tuberculosis, but it would have shed a flood of light upon much that was obscure in reference to the nature of glanders, actinomycosis, and other forms of that group of diseases now classified as the infective granulomata. Moreover, it would have been the means of leading us to discard, at an earlier period than we did, the, to us, distinctive terms "gray," "yellow," and "infiltrated" tubercle—terms which of themselves signified only the features presented at different stages of its growth of one and the same product. And even if it had not justified us in preserving a strict adherence to the expression used by myself in *The Four Bovine Scourges* to the effect "that tubercle is a visible local manifestation of a constitutional diathesis (scrofulosis)," it would at least have led to the iquiry, What is the essence of scrofula?

In order that the members of this society may the more readily realize the intimate connection which exists between animal and human tuberculosis, it is advisable that I should consider at the outset some questions of a general nature, such as the species of animals most liable to tubercle, the means by which it is propagated, the organs affected, its course, its effects on the system, its macroscopical characters, and its complications.

(1.) *Species of Animals Affected.*—It is universally acknowledged that the members of the bovine species are pre-eminently the hosts of tubercle. In them it has been thought to be indigenous, though why bovines should be more predisposed than other animals is not easy of explanation, except perhaps the fact that their complicated stomach may afford a resting place for the bacilli, or that there may be some element in the bovine constitution favorable to their support—in other words, that the doctrine of *receptivity* applies in this case more powerfully than in that of animals of other species. Looking at the fact that the sheep bites more closely to the ground than does the ox we should be inclined to think that ovines stood a greater risk of ingesting the viruliferous principle with their food than do bovines, but it is a most remarkable fact that the former are seldom found to be the subject of naturally contracted or spontaneous tuberculosis. Indeed, I may say that of all the domesticated or semi-domesticated animals whose flesh is used for human food the sheep stands out from the group as the one least liable to tubercle; and this is all the more remarkable seeing that this animal is so intimately associated in many cases with cattle and with rabbits, a creature pronouncedly predisposed to, and very largely the subject of, tuberculosis.

Koch's discovery of the tubercular bacillus has, as I have before remarked, given us a more accurate basis upon which to found a diagnosis; and several Continental pathologists, notably Johne, have within the past few years discovered its existence in the lesions of so-called lymphadenoma; while, during the past year my colleague, Professor M'Fadyean, demonstrated the bacilli in what appeared to be lymphadenomatous nodules in the spleen of the horse.

Of poultry, tuberculosis claims a holocaust of victims, and macroscopically it presents in these animals some remakable divergences from the recognized characters of tubercular products in the ox and in man. My first intimate acquaintance with avian tuberculosis was about the year 1865; but several years prior to that date I had seen cases which to the naked eye presented features closely analogous to the disease, and that more particularly in the rook.

Amongst the semi-domesticated amimals the rabbit takes the foremost place as the host of tuberculosis.

As a boy I was accustomed to see myriads of tubercular nodules in the livers of rabbits, and so much was I impressed by the fact, that in the essay already alluded to as having been read by me in 1872, I suggested that there was a possibility of contamination of pastures by the fæcal matter of these animals. Guinea-pigs are especially predisposed to tuberculosis—at least by artificial infection—and the same may be said of monkeys, in which animals the lesions approximate perhaps more closely to those seen in the human subject than they do in any other animal.

The propagation of tuberculosis is effected in a variety of ways—by congenital and hereditary transmission, by ingestion of contaminated foods or water, *i. e.*, naturally or experimentally; by natural or experimental inhalation, by accidental and intentional inoculation, and by intravenous injection.

The possibility of congenital transmission of tuberculosis occurring was for a long period denied, but there are those amongst human pathologists who believe in such a possibility, and who assert that it does occur, while veterinary pathologists have at different times adduced practical facts in proof of such a possibility. It has not as yet fallen to my lot to witness congenital tuberculosis, but I have seen it in animals at such an early age as to lead me, perforce, to the conclusion that it must have had an *intra-uterine* origin.

Mr. M'Gillivray, of Banff, has put on record evidences of the fact that congenital transmission takes place, and some six years ago I received a communication from a late pupil of my own, Mr. Frank Ashley, giving me details of some cases in a valuable herd of shorthorns, which were under the care of Mr. Carter, of Guildford, and in which young calves developed symptoms of tubercular meningitis and pulmonary tuberculosis within a few days after birth. Why doubt as to the probability of such transmission taking place should be entertained in view of our knowledge of the existence of tubercular orchitis and oöphoritis in animals, and of the existence of bacilli in the blood, with the ready transmission of the anthrax bacillus (a much larger bacillus than that of tubercle) from the mother to the fœtus, I cannot understand. Several years ago a lady of my acquaintance purchased a setting of eggs from a poultry-yard which I knew was infected, and shortly after the birth of the chickens they showed evidence of the existence of the disease, and very quickly the other poultry on the premises became affected.

As to hereditary predisposition, the fashion at the present day in human medicine seems to be to explain the fact by assuming that the systems of certain individuals—on account of family peculiarities—offer a more favorable soil for the development of the bacillus than do the systems of others. This hypothesis, to my mind, is a convenient way of explaining an unpleasant phenomenon, viz., that family after family is swept off the face of the earth by this fell malady ; but I confess I cannot understand why such a theory should be accepted in face of the many and palpable proofs to the contrary one meets with in one's intercourse with individual families.

I am well aware that the question—a most difficult one to answer—may be asked, What becomes of the virus during the years that intervene between the birth of individuals and the development of the visible manifestations of tubercular lesions in their systems? You acknowledge the possibility of syphilitic lesions being developed in children in whose systems a hereditary taint exists; is the causal entity so widely different in these diseases as to preclude the possibility of hereditary transmission in the one if you acknowledge that it can take place in the other? I grant it is difficult to understand in what particular tissue or organ the bacilli or their spores reside in a quiescent manner for prolonged periods, but when we observe the same process going on in the case of the virus of rabies, the difficulty is not so great. In the calf I have seen scrofulous lesions in the thymus gland, and scrofulous processes are common in the lymphatic glands of these animals as they are in human juveniles. May not the bacilli or their spores be imprisoned in the structure of these glands, and retain their vitality and power of reproduction until some circumstance favorable to their admission to the general system arises?

Propagation by ingestion has for long, thanks to the united labors of human and veterinary pathologists, been an established fact. Not only has the disease been propagated by the ingestion of its products, but also by the ingestion of the secretions of affected animals; and not only this, but it has been shown that a tolerably high degree of heat does not always insure the innocuity of such an important animal product as milk. This method of propagation, it has been abundantly proved, is effected between animals of different species, as from the cow to the pig, and from the former animal to the common fowl; and in addition to this, we have recently had several strong statements made as to the probability of human infection by the ingestion of improperly cooked animal food, especially that of poultry. I have already alluded to the suggestion thrown out by myself in 1872, to the effect that cattle may become infected through the medium of pastures contaminated by the fæces of tuberculous rabbits; and an observation has been made by several veterinary surgeons and by stock-owners to the effect that every animal confined in a particular and contaminated stall in particular byres became sooner or later a victim to tuberculosis. By some it has been thought that infection takes place through the medium of the lungs in these cases, but the majority of observers agree in attributing it to the ingestion, by licking (a habit common to cattle) of the dried sputa from the manger or other surroundings of infected occupants of the stalls, or by the ingestion of contaminated foods. Reference to this matter leads me to offer a remark on a very misleading statement made by human pathologists, and made by one medical man very recently (Dr. Handford in the *Lancet*), to the effect that in pulmonary phthisis of animals there is no sputum. As I pointed out in a subsequent issue of the *Lancet*, this statement is one of many exposing lamentable ignorance on the part of many medical men as to the clinical phenomena of disease in animals.

Granted that animals do not eject sputa at will, and in such unnecessary profusion as is seen too often to be the case in man, the ox and the dog do on occasion readily expectorate; and independently of this, the materials which in man would be got rid of by expectoration are passed out of the bronchial tubes by the nose, or after being coughed up into the fauces are swallowed, and ultimately passed out with the fæces to lie upon and contaminate the food, the litter, or, in the case of cattle, the pastures.

In the matter of the propagation of tubercle by the medium of the drinking water, if we acknowledge that it may be propagated by the ingestion of solid, there need be little difficulty in accepting the dictum that it may be propagated also through the medium of fluid ingesta.

At a meeting of the National Veterinary Association held in London in 1883, Mr. Olver, F. R. C. V. S., of Tamworth, expressed an opinion to the effect that a number of the cattle in his neighborhood contracted tubercle by the ingestion of sewage matter from the town.

I have myself seen one instance in which the circumstances connected with the death of a number of young cattle pointed to infection by contamination of the drinking water with sewage; and in March, 1886, I received from Mr. Menzies, M. R. C.V. S., of St. Austells, sections of the lungs of an ox affected with tuberculosis, and at the same time a communication from that gentleman in which he stated that not only the feeding bullocks, but the milch cows also on a particular farm in his neighborhood were found on examination to be the subjects of pulmonary tuberculosis; the water supply in this case was constantly contaminated by the liquid filth from the farm-yard and other places. Independently of contamination by sewage matter, both drinking water and food may become fouled by the urine and the uterine discharges of infected animals.

Propagation by inhalation has passed out of the region of speculation: it is now shown that, experimently at least, its accomplishment is certain. I do not myself see that there need be any question as to such a probability, seeing that the expired air from the lungs of tuberculous animals as well as from the lungs of man must often be laden with bacilli or their spores from the softened products of broncho-pneumonic vomicæ; but independently of this, there can be but little doubt that particles of dried sputa and other contaminated animal products, are liberated by friction and other forces into the atmosphere and become inhaled by animals.

Infection by inoculation of various products from the bodies of infected into those of healthy animals has been abundantly proved by numerous experimenters, more especially since the publication of the result of Villemin's labors; and the same may be said of subcutaneous injections, injections into cavities, and intra-venous injections; tubercular products in all stages of development and retrogression, the juice of the muscles, milk, urine, saliva, and blood have each and all been used in the successful transmission of the disease; and one experimenter (Toussaint) tells us he has succeeded in transmitting it by using the fluid developed in the vesicles of vaccinated tuberculous calves.

Probably there is no more interesting or instructive subject in connection with the malady under consideration than the transmission, re-transmission, and inter-transmission of the malady by the means above noticed. From man to nearly every species of animal, from one animal to another of the same species, and from animals of one species to others very differently constituted, has the interchange been successfully carried out; and if the resulting lesions have not always borne an absolute resemblance to their prototypes, they in this respect only stand on the same level as do the remarkable divergences in the lineaments of the countenances of closely related men and women.

The most important part, however, of this branch of experimental inquiry seems to me to lie in the probability or otherwise of the secretions from organs (healthy in themselves) becoming contaminated by the virus in the case of animals suffering from tubercular disease in other organs of the body. When general, *i. e.*, blood infection has taken place one can see that there exists more than a probability that the secretions generally will become contaminated —though this is, in my opinion at least, by no means necessary. Saliva is sometimes infective, but I have yet to see the first case of tubercular disease of any of the salivary glands. Milk is frequently infective, but I entertain (very strongly) the impression that it only becomes so when the udder is the seat of tuberculous lesions; but to this question I shall revert hereafter.

*Organs and Structures affected in Spontaneous Tubercle.*—In the ox the lungs, the liver, and the serous membranes are most largely the seat of tubercular lesions, and in reference to the abdominal and thoracic serosæ, it is thought that they are sometimes primarily affected; but I think if a careful search were made some pre-existing visceral lymphadenous focus would be found.

Meningeal tuberculosis (either cerebral or spinal) is, in my opinion, always secondary in cattle, though there are some authorities who hold that it may be produced by inhalation via the nasal chambers and the ethmoidal cells.

While allowing that serous membranes become involved by continuity or contiguity, it is to me a remarkable fact that there seems to be but little or no tendency to extension therefrom to invested organs or *vice versa.* Pounds of tubercular products are formed in and upon the peri- and the epi- cardium, upon the pleura and upon the peritoneum, without a trace thereof existing in the organs they invest; and though I do not deny that eccentric extension may take place as from the bowels, liver, or uterus, to the peritoneum, or from the lungs to the pleuræ, I have no hesitation in asserting that this method of extension is, in animals at least, extremely rare. I have seen cases of cerebral with meningeal, and of spinal with meningeal tuberculosis, but it would be difficult to assign the order of precedence in either case.

Extension from the parietes or the parenchyma of organs to mucous membranes is very common. Thus we see bronchial secondarily to pulmonary tuberculosis; but curiously enough, I have never yet seen hepatic lesions involving the mucous lining of the biliary canals, though the reverse holds good in the case of the udder and the lacteal and galactopherous sinuses.

Extension to arteries from the surrounding structures is probably never seen.

Extension to veins takes place both in the case of the pulmonary and hepatic.

Of the different glands of the body the lymphatics, especially in the ox and pig, hold pre-eminence in the frequency with which they are the seat of tuberculous lesions, and particularly does this apply to the bronchial and mediastinal groups.

The mammary glands, both of the cow and of the sow, are frequently the seat of tuberculosis, and while in the main the lesions are confined to the acini, irruption on the mucous membrane is of tolerably frequent occurrence.

Of the blood glands it may be said that while the thymus is occasionally affected, the thyroid bodies are seldom, if ever, involved; and it is a remarkable fact that the spleen is, in the ox, but very rarely the seat of tuberculous lesions, though it is more commonly affected in the pig, and, as before stated, some of the nodules we used to consider as a part of lymphadenoma in the spleen of the horse are of tubercular origin, and probably this is true also of the spleen of the dog.

Tubercular orchitis is frequently met with in the bull and in the boar, though I have not often seen it in birds. The testicles of the ox and of the boar sometimes attain an enormous size.

Tubercular oöphoritis is of common occurrence in cows; it is a frequent cause of nymphomania in these animals, and cows so afflicted are universally known as "bullers."

To tubercular metritis I have already alluded, and have remarked that the lesions seem most largely to have their origin in the sub-mucosa in which we sometimes find comparatively large cavities containing caseous matter or pus. Localization of the lesions in this organ is a frequent cause of abortion in cows, while involvement of the cervix uteri produces one form of scirrhous os.

Tubercular vaginitis is only very exceptionally seen; indeed I may say I have only met with one good instance of the lesion here.

Tubercular nephritis is also, comparatively speaking, common in cattle, the organs sometimes attaining an enormous size. Tubercular cystitis is not often seen, and when it does exist the lesion is usually localized in the cervix, while tubercular urethritis is still more rare.

Passing to the alimentary canal, we find that spontaneous tuberculosis of the mouth, the tongue, the fauces, the pharynx, and œsophagus is extremely rare, except in young calves, in which it can be readily induced, at least in the mouth, fauces, and pharynx, by the ingestion of infective milk. In the ox the three first compartments of the stomach are never affected, and the true stomach but rarely. In the stomach of the pig it is more frequently seen, and also in the crop of the fowl. The small bowels of the ox and of the pig are sometimes the seat of tubercular lesions, the large rarely; but intestinal lesions are very common in poultry and in birds, though their character differs materially from that of similar lesions in the bowels of other animals.

In the respiratory tract we find that the nasal mucous membrane and the chambers are sometimes the seat of tuberculosis in the ox. While in poultry the nostril is one of the most common sites—and the yellow, croupous-like exudate, so characteristic of the affection, is as also in the case of the mouth, pharynx, conjunctiva, and even the external aural canal, frequently confounded with and spoken of as diphtheria and croup.

In the larynx not only do we see the development of large tuberculous tumors, which are occasionally pedunculated, but we frequently see distinct ulcerations and vegetations, though one medical pathologist has gone so far as to claim that the absence of laryngeal, tracheal, and bronchial ulceration in animals is one of the characteristic differences between animal and human tuberculosis. Undoubtedly bronchial and tracheal lesions, especially ulceration, are in most cases secondary to pulmonary. They are, however, occasionally seen independently of such lesions.

In the lungs, the tubercular processes resemble in their distribution and in their characters those with which you are all familiar as occurring in the lungs of the human subject; it is sufficient for me here to say that there is not one single lesion discoverable in the lungs of man that has not its parallel in these organs in animals.

The structure of the heart is marvelously exempted from tubercular lesions, and while I have occasionally seen evidence of circumscribed interstitial lesions, I have only once seen the cardiac muscle the seat of tubercle, and even in that case the lesions were not very characteristic.

Tubercular myositis, as affecting the voluntary muscles and as an independent lesion, is of extreme rarity.

Tubercular ostitis is of comparative frequency, as is also arthritis, but the latter is in the majority of instances, secondary to the former.

Cutaneous and subcutaneous lesions are rare in mammals, except in association with the softening processes which go on in underlying organs, such as lymphatic glands, bones, and joints.

The comb and wattles of the cock, and now and then of the hen, attain an enormous size, become hard and unyielding, and on section masses of the yellow exudate already alluded to are exposed. A similar condition occurs in the foot, producing deformity of that organ, and occasionally being associated with the formation of superficial ulcers on the plantar surface, through which the softened products are discharged.

In practice, we frequently find that in pregnant cows a halt in the onward march of the disease is called, but its progress again proceeds with redoubled speed after the act of parturition; and so, too, after æstral excitement has subsided.

When acute processes are in the ascendency, the febrile state becomes marked, a temperature of 105°–107°, or even 108° Fahr., being frequently reached and maintained for days or even weeks; and as a result of this, rapid emaciation sets in and there is marked interference with the normal functions, with the exception (in many instances) of one, and that unfortunately the most important, viz., lactation.

Lastly, we not infrequently find that these acute symptoms subside, and the animal is apparently restored to perfect health and may become extremely fat. Subsequent investigation shows that the tubercular processes have been arrested or become obsolete. The clinical phenomena depend upon the organs involved.

In their macroscopical characters the tubercular lesions of the lower animals are in the main the counterpart of those with which you are familiar in man.

Milk is undoubtedly the medium through which transmission is most likely to take place; and judging from the numerous experiments which have been made, both by ingestion and inoculation, there can be little doubt as to the frequency with which it possesses viruliferous properties.

The results obtained by Professor Bang in reference to the action of heat on the bacilli are similar to those obtained by other authorities, and show how little dependence there is to be placed on the supposed disinfective properties of such a degree of heat as most people are likely to apply to milk they are about to use for dietetic purposes.

Professor M'Fadyean: The proofs of the identity of tuberculosis of man and tuberculosis of the lower animals are overwhelming. I think it may be said to divide itself naturally into—*first*, the experimental evidence; *secondly*, the histological evidence; and, *thirdly*, the evidence relating to the identity of the bacilli which modern methods enable us to discover in all tubercular growths. With reference to the first of these—the experimental evidence—I think that it is absolutely conclusive. There is but one step wanting, and that is simply that nobody has deliberately and of set purpose communicated tuberculosis to human beings by giving them tubercular flesh. With reference to the histological evidence, comparison of the minute structure of bovine tubercle with that found in the human subject certainly of itself would to me almost establish the identity of the two lesions. In particular organs of bovine animals there are types of tubercular structure that one does not often meet with in human organs. That is true principally of the chronic tubercular growth which is found so commonly in connection with the serous membranes of the bovine animals; but in these animals when an acute tuberculosis is set up, then the lesion—as, for example, the structure of the tubercle which is formed in the lung—is absolutely identical with the tubercle which is formed in the human lung. The same applies also to the tuberculosis of horses. I am not sure that an examination of the histology of the so-called tuberculosis of fowls would afford very strong evidence as to the identity of that with human or bovine tuberculosis. All the specimens that I myself possess of avian tuberculosis exhibit very considerable difference—for example, I have not in any of the specimens I have yet examined found giant cells in the lesions. With regard to the identity of the bacillus, I believe that the evidence accumulated on that head is sufficient to show that there is no recognizable difference in the characters of the tubercle bacillus when obtained from growths from bovine animals and when obtained from a human being. There is hardly any force at all in the objection which Klein urges regarding the difference of size of the tubercle bacillus as found in man and in animals. Klein himself points out that the bacillus of anthrax exhibits a very considerable difference in size when taken from different animals, and only a few pages further on he cites this difference of size of the tubercle bacillus as being evidence of the non-identity of the tubercle bacillus of men and animals. I think, then, that it must be held that it has already been abundantly proved that tuberculosis of animals and that of the human subject are identical; and it therefore is of the utmost importance to consider what are the dangers and what likelihood there is that tuberculosis of the lower animals may be communicated to human beings. That danger appears to exist, in the first place, in connection with the consumption of tubercular food, and, in the second place, in the consumption of milk from tubercular cows. I have been collecting all the udders I could get. About two years ago I procured a specimen of tuberculosis of the udder of the cow; the animal was in prime condition, and yet almost any section taken from that udder reveals the tubercle bacilli in inconceivable numbers. That cow was giving milk up till the day of slaughter, and it was not killed for disease, but because it was in a condition regarded as prime fat.

Dr. WOODHEAD: In the first place, I should like to say a few words as to the identity of the condition in man and in animals. I know that Klein insisted, as you have already heard, on certain differences between the bacilli as found in man and as found in cattle. Now, in order that I might try to convince myself, I took a series of specimens from human and bovine organs, in which the bacilli were treated in exactly the same way. I examined these specimens carefully, and I then asked several of my friends to examine them, and to tell me, if they possibly could, which was from man and which was from the cow. In almost every case it was found to be absolutely impossible to distinguish between them. Klein says that the bacilli are a third less in bovine tuberculosis than they are in man. Now, I found that it was impossible—even using very high powers—to distinguish the one from the other. There might be slight differences, but equal differences occurred in the same specimen. Then, too, when I was doing this, I also examined for the position of those bacilli, because Klein holds that there is a very distinct difference in the position of the bacilli in bovine and in human tuberculosis. He holds that the majority of bacilli are in the cells in bovine tuberculosis, and that they are between the cells in human tuberculosis. I find that other authorities have taken this up—Dr. Sutton among others, and, of course, Koch, from whom a great part of our information on this subject is derived—and these authorities distinctly say that in both human and bovine tuberculosis, you may find the bacilli both in the cells and in the spaces; and as a matter of fact, that is so. Klein, in a series of experiments reported to the Local Government Board in 1886, gives the action of bacilli taken from a human subject and inoculated into fowls; also taken from a cow and inoculated into fowls and into guinea-pigs; and also from fowls to guinea-pig; and he finds the following state of affairs—that you can inoculate fowls with tubercle taken from a human subject. In the same way you can inoculate from a guinea-pig to a cow, but when you begin to cross these—that is, when you begin to inoculate from a cow to fowls—then your experiments break down. Now, in connection with this, I should like to draw attention to one fact. Koch, in his classic experiments brought out this point very strongly —that you can cultivate tubercle bacilli at a temperature of 86° to 105°, but that beyond these limits it is exceedingly difficult to get a growth of tubercle bacilli. Now, this appears a somewhat important fact, when you come to consider the temperature of the animals on which you are experimenting. For instance, one could readily understand that you can modify the virulence, the activity of these bacilli and their power of growth. If you can modify these outside the body by temperature, you can also to a certain extent modify them by the temperature in the animals. I asked Professor M'Fadyean to-day to give me the normal temperature of some of these animals. I took a note at the time. In the cow the temperature is 101° to 102°. In the hen—and this is subject to correction—it is from 104° to 105°. In a calf about 103°; pig, same as cattle; and horse, 101°. Now, it appears that if you could only inoculate fowls with human tuberculosis—and you cannot transmit from the cow to the fowl or (probably) from the fowl to the cow—it would appear that temperature might have a very important effect on the vitality, not perhaps immediately, but on the virulent power of these bacilli. By increasing the temperature of the subject, you might diminish the power of growth and of attacking the tissues possessed by these bacilli. Of course this is only in connection with the experiments carried out, and as a partial explanation at least of what may actually take place. Then as to the transmission of tuberculosis from the bovine subject to the human subject. Principal Walley has already told us that calves are subject to tubercular ulceration of the first parts of the alimentary duct. Now, when Professor M'Fadyean and I were working at this subject, we happened to come across a series of cases in a very localized position. We had to do with a dairy in which we found evidence of tuberculosis in the udders of three cows. These three cows were giving milk, and this milk was going into a large establishment, and it was going into this establishment only. We found that in this establishment the death-rate from phthisis was in one year 40 per cent., and in another, 30 per cent. of the whole deaths. At the same time there was a series of experiments going on which were useful to us, although not so useful to the people who were carrying them on. The pigs were being fed with the milk from these same cattle, and these pigs were—a large number of them—suffering from what was evidently a tubercular condition of the lungs; and we learned that a number of these pigs were killed, and the most beautiful tubercle was found in the lungs, in the pleura, and in one or two instances in the small and large intestines. Now, these pigs were being fed on the milk from this cow-house, whereas other pigs, which otherwise were under the same conditions—the only exception being that they did not get the milk—escaped tuberculosis. These experiments were partly carried on before we went, and were continued after we left, but the pigs had the milk supply cut off simply as the result of our visit; and as a result of this, the pigs brought up in the same place afterwards escaped tuberculosis. It has been mentioned that tubercular cattle do not necessarily give tubercular milk. I think that Professor Bang has modified his position very greatly since the Copenhagen Congress, because at that time he held very strongly that it was not necessary that the udders should be tuberculous at all in order that bacilli should be found in the milk. In almost all these experiments, where tubercular infection has been brought about through tubercular milk, it has been found that the udder has been affected, whereas in those cases where the results have been negative, irregular, or doubtful, sufficient attention has not been paid to this point; and in many cases the udders were undoubtedly not tubercular. Then there is one objection which is often raised, as to the identity of tuberculosis in the human subject and in cattle. It is held that in woman tubercular mammitis is extremely rare; Dr. Cathcart has given me a reference to eight cases. In cattle it is of common occurrence. I think, however, that here we should take into account the fact that lactation in cattle is carried on under very different conditions from what it is in woman; we know that lactation is prolonged very much longer in cattle—that is, in proportion to the time required—than it is in woman, and consequently the mammary gland is under conditions certainly not favorable to health, or at least very great strength. There is practically a very great over-development of the glandular function. In connection with this I examined a series of glands from a tubercular udder, and I was astonished not only at the enormous amount of tuberculosis, but also at the very great rapidity with which it grows. With reference to the extension from the ovaries along the Fallopian tubes, it is rather curious, but true, that one month I had only six autopsies in the Sick Children's Hospital, and in three of those I found tubercular affection of the Fallopian tubes secondary to tuberculosis of the abdominal cavity. In one of these only, was there any affection in the ovary itself; in the others the tubes were affected without any affection of the ovaries.

Dr. James: The question is, in our efforts to prevent consumption, what can be done by stamping out? Now, I for one, as the result of my study, believe that the stamping out is not of the very great importance which some attach to it. In different animals, if I understood Principal Walley aright, tuberculosis is commonest in the bovine species; but in horses, I think, it is not so common. I am of opinion that it is rather due to the different lives led by these different animals. The cows are kept in the byre and the horses get plenty of exercise. The conclusion that I draw from the whole matter is, that the germ is practically ubiquitous, and when the question is put before me, are we to benefit our fellow-mortals by attempts to stamp out tuberculosis just as we can stamp out small-pox or scarlet fever? I am inclined to say that we cannot expect much in this way. I believe, as the result of my study of phthisis in man, that if you killed every tubercular animal in the country, and banished every phthisical patient, you would have as bad a set of conditions subsisting in another six months. You must deal with tuberculosis, not so much by stamping it out, as with rabies or scarlatina, but as with insanity or cancer, by improving the general conditions. The practical point, therefore, is to stamp it out to a certain extent; but to remember that our efforts would altogether fail if we did not pay more attention to the sanitary conditions of the places where they keep those animals. I believe if the byres were better looked after, and if cows, instead of being tied up in byres all day, were taken out for exercise like horses, we would be doing more to get rid of the mischief than by attempts to stamp it out, because, as I said before, the bacillus is practically ubiquitous.

